# Supplementary figures and images for: Caprine humoral response to Burkholderia pseudomallei antigens during acute melioidosis from aerosol exposure
Source: PLoS Negl Trop Dis. 2019 Feb 27;13(2):e0006851. doi: 10.1371/journal.pntd.0006851 (PMC6411198; doi:10.1371/journal.pntd.0006851)

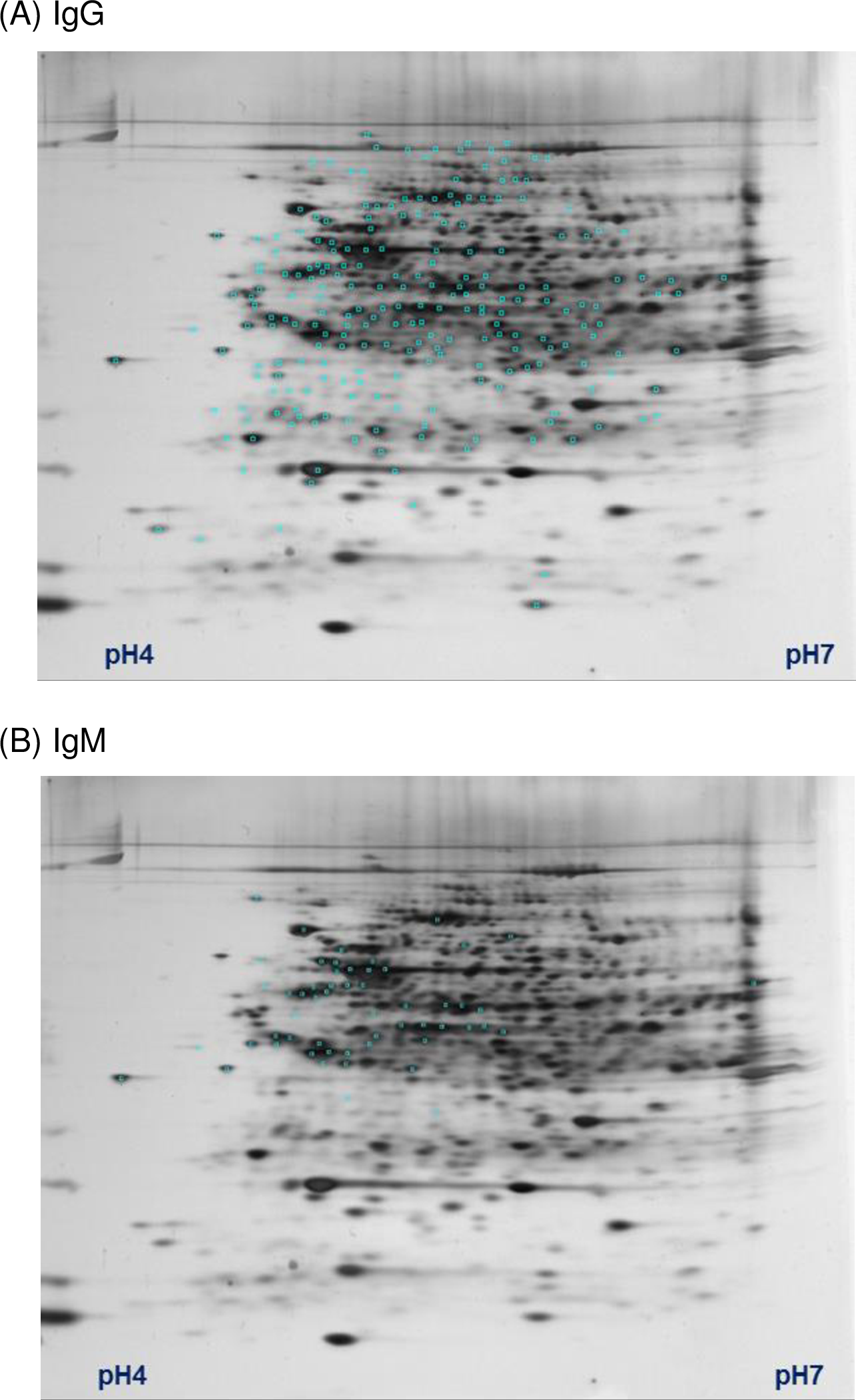

Supplement: S1 Fig — The proteins were separated by their isoelectric points followed by size separation by SDS PAGE. Cross reacting spots from western blots stained for IgG (A) and IgM (B) are mapped onto a gel stained with silver nitrate for protein. Blue marks indicate antigenic proteins that were detected for each. A total of 224 IgG reactive spots and 55 IgM reactive spots detected. (TIF) [file pntd.0006851.s001.tif]

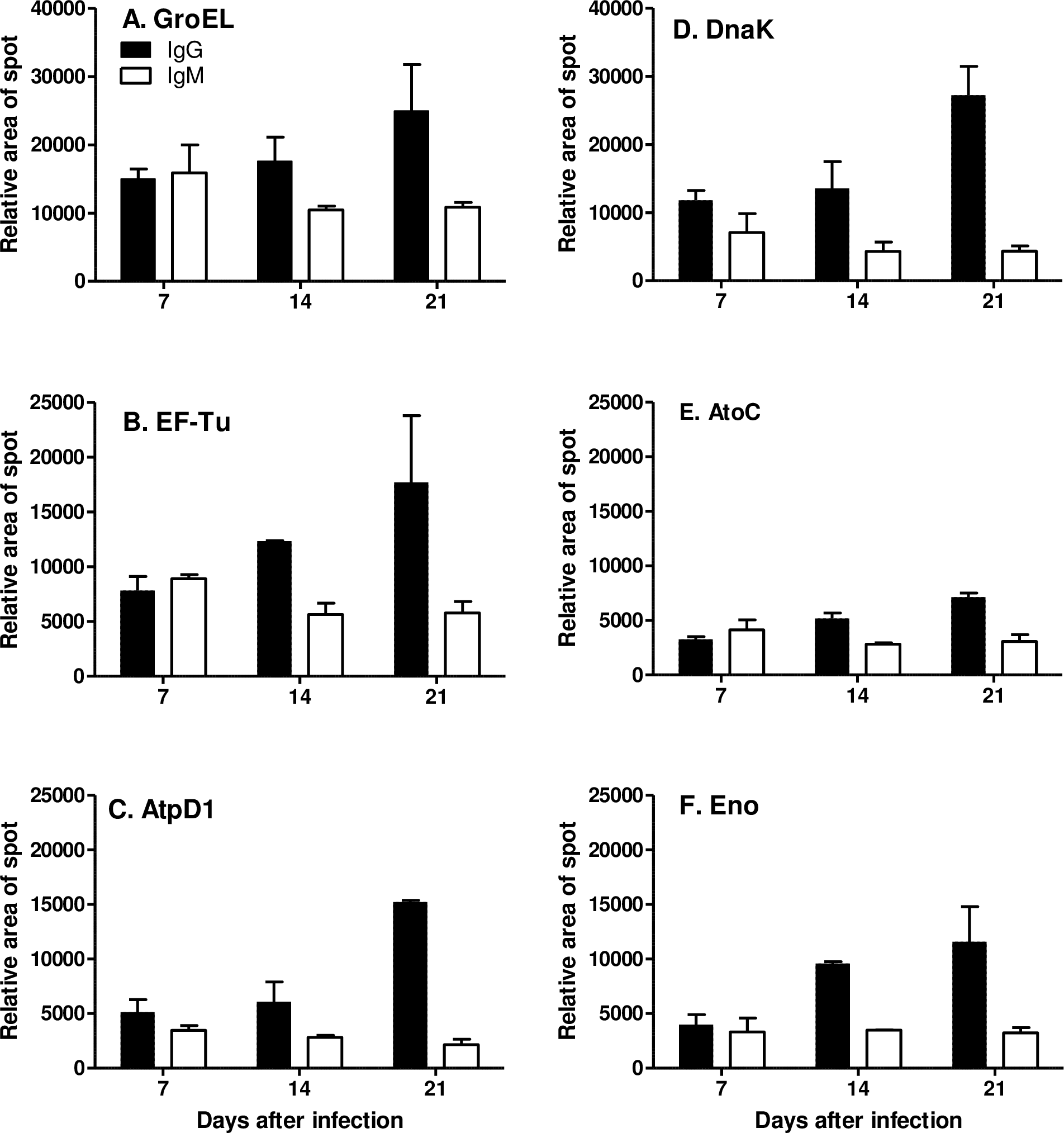

Supplement: S2 Fig — Goat humoral antibody responses of individual proteins of B. pseudomallei isolate (MSHR511). Goat sera were collected prior to challenge or on days 7, 14 and 21 after infection (note: goat sera from day 16 were included with day 14 for calculations). The intensity of goat antibody response was calculated by western blot antigenic protein spot area. (A) Heat shock protein 60 family chaperone, GroL (B) Elongation factor Tu, EF-Tu (C) ATP synthase beta chain (D) Chaperone Protein DnaK (E) Sigmal-54 dependent DNA-binding response regulator, AtoC (F) Enolase, Eno. IgG (Black bars), IgM (White bars). Immunoglobulin G (IgG, black bar), and Immunoglobulin M (IgM, white bar). (TIF) [file pntd.0006851.s002.tif]

**(A) IgG**

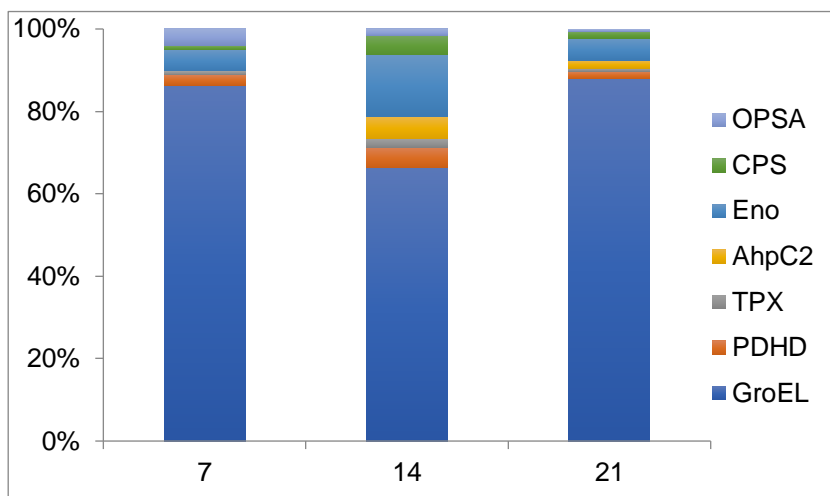

**(B) IgM**

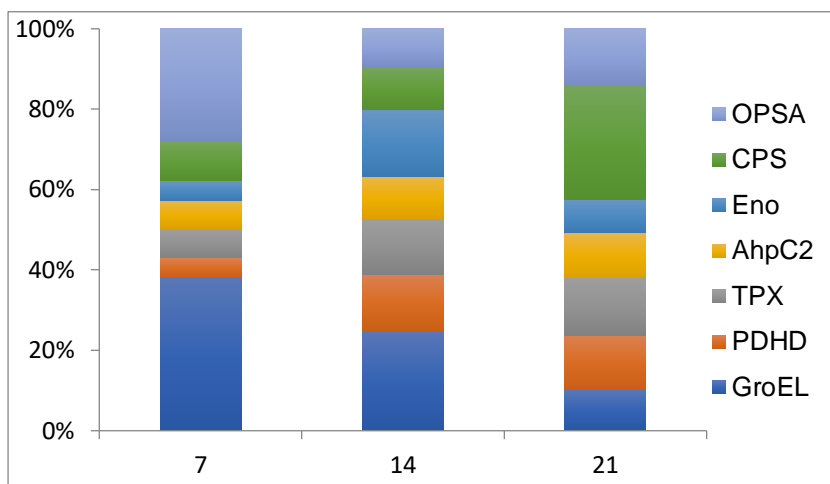

Supplement: S3 Fig — (A) Immunoglobulin IgG (IgG) antibody response was greatest for GroEL antigen for days 7, 14 and 21 followed by Eno on day 14. The humoral IgG response to the rest of the antigens (OPS A, CPS, AhpC2, TPX and PDHD) was typically <5% for each antigen. The high antibody response to GroEL antigen is thought may be due to memory B-cells being present in the immune circulation before challenge with Burkholderia pseudomallei strain MSHR511. (B) IgM antibody response was highest to GroEL antigen followed by OPS A for day 7 and these two antigens declined, respectively, for days 14 and 21. IgM response to CPS was relatively the same for days 7 and 14 but was most elevated compared to other antigens (Eno, AhpC2, TPX and PDHD) on day 21. The antibody response to the rest of the antigens (Eno, AhpC2, TPX and PDHD) was very similar. (PDF) [file pntd.0006851.s003.pdf]
