# Supplementary material for: Caprine humoral response to Burkholderia pseudomallei antigens during acute melioidosis from aerosol exposure
Source: PLoS Negl Trop Dis. 2019 Feb 27;13(2):e0006851. doi: 10.1371/journal.pntd.0006851 (PMC6411198; doi:10.1371/journal.pntd.0006851)
Supplement: S1 Table — (PDF) [file pntd.0006851.s004.pdf]

S1 Table. Primers used to amplify the genes in this study

| Gene | Locus tag | GenBank ID | Primer | Sequence (5' to 3') |
|---|---|---|---|---|
| PDHD | BPSL2299 | 53719909 | Forward | CACCATGAGTCTCATCGAAGTCAAGGT |
| | | | Reverse | TTACTTCTTGCGCTGCGG |
| TPX | BPSL2987 | 53720595 | Forward | CACCATGAGCAAAGTCACGCTGG |
| | | | Reverse | TTATTTCAGCGCGGCG |
| GroEL | BPSL2697 | 53720307 | Forward | CACCATGGCAGCTAAAGACGTCGTAT |
| | | | Reverse | TTACATGTCCATGCCCATGC |
| AhpC2 | BPSL2748 | 53720358 | Forward | CACCATGAGTCTACGTCTTGGCGAC |
| | | | Reverse | TCATTTGTTCGGCTGCG |
| Eno | BPSL2270 | 53719880 | Forward | CACCATGAGTGCAATCGTAGACATCAT |
| | | | Reverse | TCAGCGCAGATTGTAGAACG |
