# Supplementary material for: Caprine humoral response to Burkholderia pseudomallei antigens during acute melioidosis from aerosol exposure
Source: PLoS Negl Trop Dis. 2019 Feb 27;13(2):e0006851. doi: 10.1371/journal.pntd.0006851 (PMC6411198; doi:10.1371/journal.pntd.0006851)
Supplement: S2 Table — (PDF) [file pntd.0006851.s005.pdf]

S2 Table. List of purified antigens used in immunoassays

Purified antigens	Locus tag	Accession ID	Symbol	% Purity
**Recombinant Proteins**				
1. Dihydrolipoamide dehydrogenase of pyruvate dehydrogenase complex	BPSL2299	YP_108895.1	PDHD	71
2. Thiol peroxidase	BPSL2987	YP_109581.1	TPX	94
3. Heat shock protein 60 family chaperone GroEL	BPSL2697	YP_109293.1	GroEL1	60
4. Alkyl hydroperoxide reductase subunit C-like protein	BPSL2748	YP_109344.1	AhpC2	80
5. Enolase	BPSL2270	YP_108866.1	Eno	74
**Polysaccharides**				
6. Capsular polysaccharides	NA	NA	CPS	NA
7. O-polysaccharide type A	NA	NA	OPS A	NA

NA, not applicable

Recombinant proteins were purified by nickel affinity chromatography and percent purity analyzed by Image J software (http://rsb.info.nih.gov/ij/) of Sypro-Ruby (fluorescent) stained gels. Reference *B. pseudomallei* genome was strain K96243
