# Supplementary material for: Caprine humoral response to Burkholderia pseudomallei antigens during acute melioidosis from aerosol exposure
Source: PLoS Negl Trop Dis. 2019 Feb 27;13(2):e0006851. doi: 10.1371/journal.pntd.0006851 (PMC6411198; doi:10.1371/journal.pntd.0006851)
Supplement: S3 Table — (PDF) [file pntd.0006851.s006.pdf]

S3 Table. Antigenic proteins identified from an extract of *Burkholderia pseudomallei* using a non-redundant sequence database and MALDI-ToF mass spectrometry data.

**Protein function**	**Accession number**	**Theo pI** [a]	**Theo. Mw** [a]	**Total coverage (%)** [b]	**Total spectral counts** [b]	**Exclusive peptide Counts** [b]
2-methylisocitrate lyase	YP_110226.1	5.26	31.92	27	9	6
30S ribosomal protein S1	YP_109111.1	5.08	60.31	51	282	32
4-hydroxyphenylpyruvate dioxygenase	YP_109833.1	5.61	41.079	50	12	21
Acetylglutamate kinase	YP_106828.1	5.58	37.71	38	34	11
Adenylosuccinate synthase	EDU07146.1	5.72	48.30	56	41	23
Aspartate kinase, monofunctional class	YP_108834.1	5.04	45.14	32	12	10
ATP-dependent chaperone protein ClpB	ABN85130.1	5.62	95.97	75	151	67
Betaine aldehyde dehydrogenase	EDO88382.1	5.41	52.20	57	52	21
Carbamoyl-phosphate synthase, large subunit	YP_107976.1	5.12	118.24	69	229	61
Cell division protein FtsZ	YP_109616.1	4.86	41.60	19	4	3
Chain D, Crystal Structure Of S-adenosylmethionine Synthetase	3IML_D	5.13	42.94	62	28	15
Chaperonin GroL	ABA50320.1	5.13	57.15	62	129	29
Chromosome segregation protein SMC	ABN89064.1	5.13	129.01	68	199	68
Dyp-type peroxidase family protein	ABA52333.1	4.90	31.98	33	7	6
Electron transfer flavoprotein alpha-subunit	CAH36505.1	4.80	31.22	68	40	12
Elongation factor G2	YP_109810.1	5.35	76.99	68	483	39
Enolase	YP_108866.1	4.81	43.00	41	165	7
Flagellin, partial	AAD24677.1	5.05	39.16	8	4	4
Glutamyl-tRNA amidotransferase subunit B	AJX82952.1	5.31	53.32	60	39	21
Glutathione-disulfide reductase	YP_106924.1	5.72	48.20	58	26	17
Histidine--tRNA ligase	EIF52704.1	5.06	49.60	62	22	18
Hsp33 family protein	ABA48351.1	5.17	34.96	41	14	10
Hypothetical protein (DUF2957)	CAH36714.1	4.97	54.63	38	60	13
Hypothetical protein DP65_6045	KGC58800.1	5.85	124.16	19	17	16

Hypothetical protein JE55_2844	AIP79200.1	5.16	43.09	51	22	14
Molecular chaperone GroES	YP_109294.1	5.79	10.74	62	11	5
nlpB/DapX lipofamily protein	KGC74851.1	6.15	41.21	64	65	1
Phenylacetic acid degradation protein paaN	ABA48915.1	5.73	60.66	51	50	22
Phosphoglucomutase	WP_004546269.1	5.28	56.50	56	92	25
Sigma-54 dependent DNA-binding response regulator	CAH39735.1	5.06	51.07	47	39	20
Succinate-CoA ligase, beta subunit	YP_107404.1	5.25	41.30	53	31	17
Survival protein SurA precursor	YP_107288.1	7.84	49.14	49	31	30
Universal stress family protein	AFI69175.1	5.79	30.46	21	5	5

[a] Theoretical molecular weight and isoelectric point were determined using Compute pI/MW tool at ExPASy.

[b] Mass spectrometry results. Proteins were identified by MALDI-ToF mass spectrometry. The numbers given are the coverage of the target protein sequence by mass spec identified peptides, Total spectral counts equal the number of peptides detected by the mass spectrometer, and exclusive peptides sequences, which indicates the peptide sequences that includes non-overlapped sequences.
