# Supplementary material for: Caprine humoral response to Burkholderia pseudomallei antigens during acute melioidosis from aerosol exposure
Source: PLoS Negl Trop Dis. 2019 Feb 27;13(2):e0006851. doi: 10.1371/journal.pntd.0006851 (PMC6411198; doi:10.1371/journal.pntd.0006851)
Supplement: S4 Table — (PDF) [file pntd.0006851.s007.pdf]

S4 Table. Immunity (immune frequency) of antigenic proteins over days after infection.

Protein function	Pre-infection		7 Days after infection		14 Days after infection		21 Days after infection	
	IgM 8 sera tested	IgG 8 tested	IgM 3 tested	IgG 3tested	IgM 3tested	IgG 3tested	IgM 2tested	IgG 2tested
Translation elongation factor Tu	4[a]	7	3[b]	3	3	3	2	2
Heat shock protein 60 family chaperone GroEL	4	8	3	3	2	3	2	2
Chaperone protein DnaK	3	8	3	3	3	3	2	2
Sigma-54 dependent DNA-binding response regulator	3	7	3	3	3	3	2	2
ATP synthase beta chain (EC 3.6.3.14)	3	2	3	3	3	3	2	2
Unidentified protein (M3-7306)[c]	3	4	1	3	3	3	1	2
Translation elongation factor Tu	3	1	0	1	2	2	1	2
Unidentified protein (M3-7631)	2	4	3	3	3	3	1	2
Cell division trigger factor	2	2	3	2	3	3	1	2
Unidentified protein (M3-7630)	2	1	2	2	3	3	1	1
Dihydrolipoamide dehydrogenase	2	0	2	0	3	2	1	0
Cell division protein FtsA	2	4	0	1	3	1	1	0
Enolase (EC 4.2.1.11)	1	1	1	1	3	2	2	2
Unidentified protein (M3-7420)	1	3	3	2	2	3	1	2
Unidentified protein (M3-7216)	1	1	0	2	2	2	1	2
Electron transfer flavoprotein, alpha subunit	1	0	2	1	2	2	1	2
Unidentified protein (M3-7401)	1	0	1	0	2	2	0	2
Unidentified protein (M5-0813)	1	0	1	2	0	2	0	1
Unidentified protein (M3-7632)	1	0	1	0	1	1	0	1
Unidentified protein (M3-7268)	1	0	0	0	2	0	1	0
Unidentified protein (M3-7107)	1	0	0	0	0	0	1	0
Unidentified protein (M5-0489)	0	4	1	3	2	3	1	2
Unidentified protein (M5-0490)	0	4	1	3	2	3	1	2
Elongation factor G2	0	1	0	1	0	3	1	2

Unidentified protein (M3-7328)	0	0	2	2	2	2	1	2
Unidentified protein (M3-7633)	0	1	0	1	1	1	1	2
RecA protein	0	0	0	2	0	1	1	2
Unidentified protein (M3-7381)	0	1	0	2	0	3	0	2
Unidentified protein (M3-7476)	0	0	0	2	0	3	0	2
Unidentified protein (M5-0233)	0	5	0	1	0	3	0	2
Unidentified protein (M3-7479)	0	2	0	0	0	3	0	2
Unidentified protein (M3-7628)	0	0	0	0	0	3	0	2
Unidentified protein (M3-7430)	0	0	0	0	0	3	0	2
Unidentified protein (M3-7076)	0	1	0	1	0	2	0	2
Alkyl hydroperoxide reductase protein C	0	1	0	0	0	2	0	2
Unidentified protein (M3-7496)	0	0	0	0	0	2	0	2
Unidentified protein (M5-0433)	0	0	0	1	0	1	0	2
Unidentified protein (M5-0229)	0	1	0	0	0	1	0	2
Autotransporter adhesin	0	0	0	0	0	1	0	2
Unidentified protein (M3-7498)	0	0	0	0	0	1	0	2
Unidentified protein (M3-7288)	0	0	0	1	0	0	0	2
Unidentified protein (M5-0116)	0	0	0	0	0	0	0	2
Unidentified protein (M3-7217)	0	0	0	0	0	0	0	2
Unidentified protein (M3-7302)	0	0	0	0	0	0	0	2
Unidentified protein (M3-7414)	0	0	0	0	0	0	0	2
Unidentified protein (M3-7470)	0	0	0	0	0	0	0	2
Unidentified protein (M3-7468)	0	0	0	0	0	0	0	2
Unidentified protein (M5-W274)	0	0	0	0	0	0	0	2
Unidentified protein (M3-7424)	0	0	0	0	0	0	0	2
Unidentified protein (M3-7446)	0	0	0	0	0	0	0	2
Unidentified protein (M3-7307)	0	0	0	2	1	3	1	1
Unidentified protein (M3-7353)	0	0	0	0	0	3	1	1
Unidentified protein (M3-7644)	0	0	0	0	1	2	1	1
Unidentified protein (M3-7388)	0	0	1	3	2	1	1	1
Unidentified protein (M3-7384)	0	0	0	2	1	1	1	1

Unidentified protein (M5-0310)	0	0	1	0	0	1	1	1
Unidentified protein (M5-0416)	0	0	0	0	0	1	1	1
Unidentified protein (M5-0343)	0	1	1	2	0	3	0	1
Unidentified protein (M5-0512)	0	1	0	0	0	3	0	1
Unidentified protein (M3-7647)	0	0	0	0	0	3	0	1
Unidentified protein (M3-7090)	0	0	0	0	0	3	0	1
Unidentified protein (M3-7323)	0	0	0	2	0	2	0	1
Unidentified protein (M3-7028)	0	1	0	1	0	2	0	1
Unidentified protein (M5-0642)	0	0	0	1	0	2	0	1
Unidentified protein (M3-7075)	0	1	0	0	0	2	0	1
Unidentified protein (M3-7074)	0	0	0	0	0	2	0	1
Unidentified protein (M3-7058)	0	0	0	0	0	2	0	1
Unidentified protein (M5-0375)	0	0	0	0	0	2	0	1
Succinylarginine dihydrolase	0	0	0	0	0	2	0	1
Unidentified protein (M3-7410)	0	0	0	0	0	2	0	1
Unidentified protein (M5-0565)	0	0	0	0	0	2	0	1
Unidentified protein (M5-0336)	0	0	0	0	1	1	0	1
Unidentified protein (M3-7346)	0	0	0	0	1	1	0	1
Unidentified protein (M5-0533)	0	0	0	0	1	1	0	1
TldE/PmbA protein	0	0	0	1	0	1	0	1
Unidentified protein (M3-7473)	0	0	0	1	0	1	0	1
Granule-associated protein	0	0	0	1	0	1	0	1
Unidentified protein (M3-7324)	0	0	1	0	0	1	0	1
chromosome segregation protein SMC	0	0	0	0	0	1	0	1
Unidentified protein (M3-6998)	0	0	0	0	0	1	0	1
Unidentified protein (M3-7036)	0	0	0	0	0	1	0	1
Unidentified protein (M3-7073)	0	0	0	0	0	1	0	1
Unidentified protein (M5-0183)	0	0	0	0	0	1	0	1
Unidentified protein (M3-7127)	0	0	0	0	0	1	0	1
Unidentified protein (M3-7154)	0	0	0	0	0	1	0	1
Hypothetical protein	0	0	0	0	0	1	0	1

Unidentified protein (M5-0438)	0	0	0	0	0	1	0	1
Unidentified protein (M5-0452)	0	0	0	0	0	1	0	1
Unidentified protein (M5-0464)	0	0	0	0	0	1	0	1
Unidentified protein (M5-0478)	0	0	0	0	0	1	0	1
Unidentified protein (M3-7357)	0	0	0	0	0	1	0	1
Unidentified protein (M3-7372)	0	0	0	0	0	1	0	1
Unidentified protein (M3-7366)	0	0	0	0	0	1	0	1
nlpB/DapX lipofamily protein	0	0	0	0	0	1	0	1
Unidentified protein (M5-0542)	0	0	0	0	0	1	0	1
Unidentified protein (M5-0702)	0	0	0	0	0	1	0	1
Unidentified protein (M3-7551)	0	0	0	0	0	1	0	1
Unidentified protein (M3-7423)	0	0	0	0	0	1	0	1
Unidentified protein (M5-W292)	0	0	0	0	0	1	0	1
Unidentified protein (M5-0279)	0	0	0	0	1	0	0	1
ATP-dependent Clp protease ATP-binding subunit Clp	0	0	0	0	1	0	0	1
S-adenosylmethionine synthetase	0	0	0	0	1	0	0	1
succinate-CoA ligase, beta subunit	0	0	0	0	1	0	0	1
Unidentified protein (M5-0821)	0	0	0	1	0	0	0	1
Unidentified protein (M5-W201)	0	0	0	1	0	0	0	1
Unidentified protein (M5-0585)	0	0	1	0	0	0	0	1
S-adenosylmethionine synthetase	0	1	0	0	0	0	0	1
Unidentified protein (M3-7650)	0	1	0	0	0	0	0	1
carbamoyl-phosphate synthase, large subunit	0	0	0	0	0	0	0	1
Unidentified protein (M5-0055)	0	0	0	0	0	0	0	1
Unidentified protein (M3-6927)	0	0	0	0	0	0	0	1
Unidentified protein (M5-0070)	0	0	0	0	0	0	0	1
Unidentified protein (M5-0075)	0	0	0	0	0	0	0	1
Unidentified protein (M3-6957)	0	0	0	0	0	0	0	1
Unidentified protein (M3-6958)	0	0	0	0	0	0	0	1
Unidentified protein (M5-0083)	0	0	0	0	0	0	0	1
Unidentified protein (M5-0085)	0	0	0	0	0	0	0	1

Unidentified protein (M3-6965)	0	0	0	0	0	0	0	1
Unidentified protein (M5-0090)	0	0	0	0	0	0	0	1
Unidentified protein (M5-0094)	0	0	0	0	0	0	0	1
Unidentified protein (M5-0108)	0	0	0	0	0	0	0	1
Unidentified protein (M3-7033)	0	0	0	0	0	0	0	1
Unidentified protein (M3-7021)	0	0	0	0	0	0	0	1
Unidentified protein (M3-7083)	0	0	0	0	0	0	0	1
Unidentified protein (M3-7114)	0	0	0	0	0	0	0	1
Unidentified protein (M5-0259)	0	0	0	0	0	0	0	1
Unidentified protein (M3-7641)	0	0	0	0	0	0	0	1
Unidentified protein (M5-0269)	0	0	0	0	0	0	0	1
Carboxypeptidase C (cathepsin A)	0	0	0	0	0	0	0	1
Unidentified protein (M3-7252)	0	0	0	0	0	0	0	1
Unidentified protein (M5-0368)	0	0	0	0	0	0	0	1
Unidentified protein (M5-0388)	0	0	0	0	0	0	0	1
Unidentified protein (M5-0393)	0	0	0	0	0	0	0	1
Unidentified protein (M3-7266)	0	0	0	0	0	0	0	1
Dihyrolipoamide dehydrogenase	0	0	0	0	0	0	0	1
Unidentified protein (M3-7281)	0	0	0	0	0	0	0	1
Hypothetical protein DP56_1416	0	0	0	0	0	0	0	1
Survival protein SurA precursor	0	0	0	0	0	0	0	1
Unidentified protein (M3-7311)	0	0	0	0	0	0	0	1
Unidentified protein (M3-7302)	0	0	0	0	0	0	0	1
Unidentified protein (M3-7371)	0	0	0	0	0	0	0	1
Unidentified protein (M3-7382)	0	0	0	0	0	0	0	1
DNA-directed RNA polymerase alpha subunit	0	0	0	0	0	0	0	1
Unidentified protein (M5-0528)	0	0	0	0	0	0	0	1
Unidentified protein (M3-7387)	0	0	0	0	0	0	0	1
Unidentified protein (M3-7402)	0	0	0	0	0	0	0	1
Flagellar biosynthesis protein FliC	0	0	0	0	0	0	0	1
Unidentified protein (M5-0558)	0	0	0	0	0	0	0	1

Unidentified protein (M3-7408)	0	0	0	0	0	0	0	1
Unidentified protein (M3-7418)	0	0	0	0	0	0	0	1
Unidentified protein (M3-7422)	0	0	0	0	0	0	0	1
Unidentified protein (M5-0568)	0	0	0	0	0	0	0	1
Unidentified protein (M5-0569)	0	0	0	0	0	0	0	1
Unidentified protein (M3-7428)	0	0	0	0	0	0	0	1
Unidentified protein (M5-0574)	0	0	0	0	0	0	0	1
Unidentified protein (M5-0577)	0	0	0	0	0	0	0	1
Unidentified protein (M5-0581)	0	0	0	0	0	0	0	1
Unidentified protein (M3-7434)	0	0	0	0	0	0	0	1
Unidentified protein (M3-7433)	0	0	0	0	0	0	0	1
Unidentified protein (M3-7453)	0	0	0	0	0	0	0	1
Unidentified protein (M3-7437)	0	0	0	0	0	0	0	1
Unidentified protein (M3-7441)	0	0	0	0	0	0	0	1
Unidentified protein (M5-0600)	0	0	0	0	0	0	0	1
Unidentified protein (M3-7450)	0	0	0	0	0	0	0	1
Unidentified protein (M5-0602)	0	0	0	0	0	0	0	1
Acetylglutamate kinase	0	0	0	0	0	0	0	1
Unidentified protein (M5-0608)	0	0	0	0	0	0	0	1
Unidentified protein (M3-7467)	0	0	0	0	0	0	0	1
Unidentified protein (M5-0633)	0	0	0	0	0	0	0	1
Unidentified protein (M3-7471)	0	0	0	0	0	0	0	1
Unidentified protein (M5-0640)	0	0	0	0	0	0	0	1
Unidentified protein (M3-7481)	0	0	0	0	0	0	0	1
Unidentified protein (M5-0644)	0	0	0	0	0	0	0	1
Unidentified protein (M3-7477)	0	0	0	0	0	0	0	1
Unidentified protein (M3-7482)	0	0	0	0	0	0	0	1
Unidentified protein (M5-0654)	0	0	0	0	0	0	0	1
Unidentified protein (M3-7500)	0	0	0	0	0	0	0	1
Unidentified protein (M3-7503)	0	0	0	0	0	0	0	1
Unidentified protein (M5-0670)	0	0	0	0	0	0	0	1

Unidentified protein (M3-7505)	0	0	0	0	0	0	0	1
Unidentified protein (M3-7512)	0	0	0	0	0	0	0	1
Unidentified protein (M3-7514)	0	0	0	0	0	0	0	1
Unidentified protein (M3-7567)	0	0	0	0	0	0	0	1
Unidentified protein (M5-0767)	0	0	0	0	0	0	0	1
Unidentified protein (M3-7592)	0	0	0	0	0	0	0	1
Unidentified protein (M5-0810)	0	0	0	0	0	0	0	1
Unidentified protein (M5-0822)	0	0	0	0	0	0	0	1
Unidentified protein (M5-0824)	0	0	0	0	0	0	0	1
Unidentified protein (M5-0826)	0	0	0	0	0	0	0	1
Unidentified protein (M5-0828)	0	0	0	0	0	0	0	1
Unidentified protein (M5-W016)	0	0	0	0	0	0	0	1
Unidentified protein (M5-W017)	0	0	0	0	0	0	0	1
Unidentified protein (M3-6883)	0	0	0	0	0	0	0	1
Unidentified protein (M5-W187)	0	0	0	0	0	0	0	1
Unidentified protein (M3-6898)	0	0	0	0	0	0	0	1
Unidentified protein (M3-6903)	0	0	0	0	0	0	0	1
Unidentified protein (M3-6888)	0	0	0	0	0	0	0	1
Unidentified protein (M5-W225)	0	0	0	0	0	0	0	1
Unidentified protein (M5-W228)	0	0	0	0	0	0	0	1
Unidentified protein (M3-6889)	0	0	0	0	0	0	0	1
Unidentified protein (M3-7312)	0	0	0	0	0	0	0	1
Unidentified protein (M3-7391)	0	0	0	0	0	0	0	1
Unidentified protein (M5-W030)	0	0	0	0	0	0	0	1
Unidentified protein (M5-W308)	0	0	0	0	0	0	0	1
Unidentified protein (M5-W031)	0	0	0	0	0	0	0	1
Unidentified protein (M5-W322)	0	0	0	0	0	0	0	1
Unidentified protein (M5-W033)	0	0	0	0	0	0	0	1
Unidentified protein (M5-W354)	0	0	0	0	0	0	0	1
Unidentified protein (M5-W359)	0	0	0	0	0	0	0	1
Unidentified protein (M5-W371)	0	0	0	0	0	0	0	1

Unidentified protein (M5-W412)	0	0	0	0	0	0	0	1
Unidentified protein (M5-W414)	0	0	0	0	0	0	0	1
Unidentified protein (M5-W051)	0	0	0	0	0	0	0	1
Unidentified protein (M5-W053)	0	0	0	0	0	0	0	1
Unidentified protein (M3-7026)	0	0	0	0	0	0	0	1
Unidentified protein (M3-7018)	0	0	0	0	0	0	1	0
30S ribosomal protein S1	0	2	0	0	1	3	0	0
Unidentified protein (M3-7128)	0	0	0	0	0	2	0	0
Unidentified protein (M3-7143)	0	0	0	0	0	2	0	0
ATP synthase alpha chain (EC 3.6.3.14)	0	0	0	0	0	2	0	0
Unidentified protein (M5-0401)	0	0	0	0	0	2	0	0
Unidentified protein (M3-7165)	0	0	0	0	1	1	0	0
ATP synthase beta chain (EC 3.6.3.14)	0	0	0	1	0	1	0	0
Unidentified protein (M3-7645)	0	0	0	1	0	1	0	0
Succinylarginine dihydrolase	0	0	0	1	0	1	0	0
Unidentified protein (M3-7327)	0	0	0	1	0	1	0	0
Unidentified protein (M5-0517)	0	0	0	1	0	1	0	0
Unidentified protein (M3-7117)	0	1	0	0	0	1	0	0
Translation elongation factor Tu	0	1	0	0	0	1	0	0
Unidentified protein (M3-6962)	0	0	0	0	0	1	0	0
Unidentified protein (M5-0192)	0	0	0	0	0	1	0	0
Unidentified protein (M5-0196)	0	0	0	0	0	1	0	0
Unidentified protein (M5-0203)	0	0	0	0	0	1	0	0
Unidentified protein (M3-7118)	0	0	0	0	0	1	0	0
Unidentified protein (M5-0219)	0	0	0	0	0	1	0	0
Unidentified protein (M3-7122)	0	0	0	0	0	1	0	0
Unidentified protein (M3-7179)	0	0	0	0	0	1	0	0
Unidentified protein (M3-7208)	0	0	0	0	0	1	0	0
Unidentified protein (M3-7178)	0	0	0	0	0	1	0	0
Unidentified protein (M3-7193)	0	0	0	0	0	1	0	0
Unidentified protein (M3-7186)	0	0	0	0	0	1	0	0

Unidentified protein (M3-7239)	0	0	0	0	0	1	0	0
Unidentified protein (M5-0360)	0	0	0	0	0	1	0	0
Unidentified protein (M3-7241)	0	0	0	0	0	1	0	0
Unidentified protein (M5-0374)	0	0	0	0	0	1	0	0
Unidentified protein (M3-7230)	0	0	0	0	0	1	0	0
Unidentified protein (M3-7237)	0	0	0	0	0	1	0	0
Isovaleryl-CoA dehydrogenase	0	0	0	0	0	1	0	0
Unidentified protein (M3-7293)	0	0	0	0	0	1	0	0
Unidentified protein (M3-7310)	0	0	0	0	0	1	0	0
Unidentified protein (M3-7330)	0	0	0	0	0	1	0	0
Unidentified protein (M3-7340)	0	0	0	0	0	1	0	0
Unidentified protein (M3-7336)	0	0	0	0	0	1	0	0
Unidentified protein (M3-7364)	0	0	0	0	0	1	0	0
Unidentified protein (M3-7389)	0	0	0	0	0	1	0	0
Unidentified protein (M3-7466)	0	0	0	0	0	1	0	0
Unidentified protein (M5-0705)	0	0	0	0	0	1	0	0
Unidentified protein (M5-0715)	0	0	0	0	0	1	0	0
Unidentified protein (M5-0817)	0	0	0	0	0	1	0	0
Unidentified protein (M3-7635)	0	0	0	0	0	1	0	0
Unidentified protein (M5-W114)	0	0	0	0	0	1	0	0
Unidentified protein (M3-7283)	0	0	0	0	0	1	0	0
Unidentified protein (M5-W165)	0	0	0	0	0	1	0	0
Unidentified protein (M5-W164)	0	0	0	0	0	1	0	0
Unidentified protein (M5-W086)	0	0	0	0	0	1	0	0
Unidentified protein (M5-W015)	0	0	0	0	0	1	0	0
Unidentified protein (M3-7092)	0	0	0	0	0	1	0	0
Unidentified protein (M3-7285)	0	0	0	0	1	0	0	0
histidine-tRNA ligase	0	0	0	1	0	0	0	0
Unidentified protein (M5-0501)	0	0	0	1	0	0	0	0
Unidentified protein (M3-7497)	0	0	0	1	0	0	0	0
Unidentified protein (M3-7099)	0	0	1	0	0	0	0	0

Unidentified protein (M3-7125)	0	0	1	0	0	0	0	0
Unidentified protein (M5-0282)	0	0	1	0	0	0	0	0
Unidentified protein (M5-0331)	0	0	1	0	0	0	0	0
Unidentified protein (M3-7278)	0	0	1	0	0	0	0	0
Unidentified protein (M3-7439)	0	0	1	0	0	0	0	0
Unidentified protein (M5-0823)	0	0	1	0	0	0	0	0
Unidentified protein (M3-7424)	0	0	1	0	0	0	0	0
Unidentified protein (M3-W005)	0	1	0	0	0	0	0	0

[a] 3 : Three detected immunogenic spot number of 8 pre-infection western blot.

[b] 3 : Three detected immunogenic spot number of 3 spots of infected goat serum western blot.

[c] Unidentified protein (M3-7306) is one of unidentified protein for protein function but detected by western blotting.
